# Supplementary material for: SCOPE enables type III CRISPR-Cas diagnostics using flexible targeting and stringent CARF ribonuclease activation
Source: Nat Commun. 2021 Aug 19;12:5033. doi: 10.1038/s41467-021-25337-5 (PMC8376896; doi:10.1038/s41467-021-25337-5)
Supplement: Supplementary file 3 — Reporting Summary [file 41467_2021_25337_MOESM3_ESM.pdf]

nature portfolio

Corresponding author(s): Raymond H.J. Staals and Yifan Zhu

Last updated by author(s): Jul 28, 2021

Reporting Summary

Nature Portfolio wishes to improve the reproducibility of the work that we publish. This form provides structure for consistency and transparency in reporting. For further information on Nature Portfolio policies, see our Editorial Policies and the Editorial Policy Checklist.

Statistics

For all statistical analyses, confirm that the following items are present in the figure legend, table legend, main text, or Methods section.

n/a	Confirmed	
☐	☒	The exact sample size (*n*) for each experimental group/condition, given as a discrete number and unit of measurement
☐	☒	A statement on whether measurements were taken from distinct samples or whether the same sample was measured repeatedly
☒	☐	The statistical test(s) used AND whether they are one- or two-sided<br>*Only common tests should be described solely by name; describe more complex techniques in the Methods section.*
☒	☐	A description of all covariates tested
☒	☐	A description of any assumptions or corrections, such as tests of normality and adjustment for multiple comparisons
☐	☒	A full description of the statistical parameters including central tendency (e.g. means) or other basic estimates (e.g. regression coefficient) AND variation (e.g. standard deviation) or associated estimates of uncertainty (e.g. confidence intervals)
☒	☐	For null hypothesis testing, the test statistic (e.g. *F*, *t*, *r*) with confidence intervals, effect sizes, degrees of freedom and *P* value noted<br>*Give P values as exact values whenever suitable.*
☒	☐	For Bayesian analysis, information on the choice of priors and Markov chain Monte Carlo settings
☒	☐	For hierarchical and complex designs, identification of the appropriate level for tests and full reporting of outcomes
☒	☐	Estimates of effect sizes (e.g. Cohen's *d*, Pearson's *r*), indicating how they were calculated

Our web collection on statistics for biologists contains articles on many of the points above.

Software and code

Policy information about availability of computer code

Data collection	Data collection regarding the structural analyses presented in Figure 4 was conducted and described in a previous study (Taylor et al., Science 2015). The BioTek Gen5 (v3.11) software was used for the cOA quantification presented in Figures 2C,F and 3C,F,I. Data presented in Figure 5B,D,F were collected with CFX Maestro Software (v2.2) and Figure 5E with 7500 Fast SDS (v1.4.1). Results from the qPCR (Table S2) were collected with the 7500 Fast SDS (v1.4.1) software.
Data analysis	Reanalyses of the structural models (Figure 4) was performed with ChimeraX (v1.1) using the Isolde (v1.0b3) plugin (https://isolde.cimr.cam.ac.uk/)

For manuscripts utilizing custom algorithms or software that are central to the research but not yet described in published literature, software must be made available to editors and reviewers. We strongly encourage code deposition in a community repository (e.g. GitHub). See the Nature Portfolio guidelines for submitting code & software for further information.

Data

Policy information about availability of data

All manuscripts must include a data availability statement. This statement should provide the following information, where applicable:

- Accession codes, unique identifiers, or web links for publicly available datasets
- A description of any restrictions on data availability
- For clinical datasets or third party data, please ensure that the statement adheres to our policy

The structures of the different TtCmr complexes (Figure 4) have previously been deposited into the EMDataBank with accession codes EMD-2898 (https://www.ebi.ac.uk/pdbe/entry/emdb/EMD-2898) and EMD-2899(https://www.ebi.ac.uk/pdbe/entry/emdb/EMD-2899) (Taylor et al., Science 2015). All other data from this study is available from the manuscript, the supplementary information or from the associated Source Data

Field-specific reporting

Please select the one below that is the best fit for your research. If you are not sure, read the appropriate sections before making your selection.

- [x] Life sciences
- [ ] Behavioural & social sciences
- [ ] Ecological, evolutionary & environmental sciences

For a reference copy of the document with all sections, see nature.com/documents/nr-reporting-summary-flat.pdf

Life sciences study design

All studies must disclose on these points even when the disclosure is negative.

Sample size	No sample-size (determination) calculation was applied in this study. In order to obtain statistic significances, we choose a sample sizes of 3 for the in vitro experiments based on standard practices in the field.
Data exclusions	No data was excluded in this study
Replication	All of the depicted gels (Figure 1B, Figure 2B,E, Figure 3B,E,H, Figure 5A, Figure S3) have been successfully been repeated at least 3 times (with the exception of Figure 1C, which has been repeated 2 times) with similar outcomes. cOA production assays (Figure 2C,F, Figure 3C,F,I, Figure S1, Figure S2) were repeated in triplicate. The experiments depicted in Figures 5B,D,F were performed in triplicate. The data depicted in Figure 5E, Table S2 and Figure S5 were obtained from single measurements due to the limited availability of the human swap samples.
Randomization	No randomization was required/applied, as this cannot be applied to our biochemical vitro assays nor to structural analyses as presented in this study.
Blinding	Blinding of the human swap samples (Figure 5E) was not applied, as the samples were unknown to be either positive or negative for SARS-CoV2 prior to the SCOPE test and qPCR validation was performed independently afterwards. Blinding is not relevant to any of the other (in vitro) experiments presented in the study, as these were based on predefined substrates and enzymes.

Reporting for specific materials, systems and methods

We require information from authors about some types of materials, experimental systems and methods used in many studies. Here, indicate whether each material, system or method listed is relevant to your study. If you are not sure if a list item applies to your research, read the appropriate section before selecting a response.

Materials & experimental systems

n/a	Involved in the study	
[x]	[ ]	Antibodies
[x]	[ ]	Eukaryotic cell lines
[x]	[ ]	Palaeontology and archaeology
[x]	[ ]	Animals and other organisms
[ ]	[x]	Human research participants
[x]	[ ]	Clinical data
[x]	[ ]	Dual use research of concern

Methods

n/a	Involved in the study	
[x]	[ ]	ChIP-seq
[x]	[ ]	Flow cytometry
[x]	[ ]	MRI-based neuroimaging

Human research participants

Policy information about studies involving human research participants

Population characteristics	Adults with either SARS-CoV-2- related symptoms or who had contact with infected persons, who visited the COVID-19 community testing center, located at the University Medical Center Utrecht (UMCU) in the Netherlands.
Recruitment	Recruitment occurred at a testing site that was set up as a research site to evaluate new diagnostic tests for SARS-CoV-2. Adults who visited the testing center were asked to participate in validation studies of novel diagnostic tests. Participants gave consent to use of residual material of the obtained combined human throat/nasopharyngeal swab samples.
Ethics oversight	The medical research ethics committee (MREC) of Utrecht decided that validation studies of new diagnostic tests for SARS-CoV-2 is not subject to the Medical Research Involving Human Subjects Act (WMO) and did not require full review by an accredited MREC. All participants were informed and consented with participation.

Note that full information on the approval of the study protocol must also be provided in the manuscript.
